# Comprehensive analysis of cefuroxime-associated adverse events: Insights from JADER database (2004–2024) and comparison with FAERS database

**DOI:** 10.1097/MD.0000000000049232

**Published:** 2026-06-12

**Authors:** Fangqiong Li, Jinyu Luo, Xinhao Zhang, Cheng Jiang, Guoqin Xia

**Affiliations:** a Department of Clinical Laboratory, Tongde Hospital of Zhejiang Province, Hangzhou, Zhejiang, China; b Zhejiang Academy of Traditional Chinese Medicine, Tongde Hospital of Zhejiang Province Affiliated to Zhejiang Chinese Medical University, Hangzhou, Zhejiang, China; c Zhejiang Key Laboratory of Disease-Syndrome Integrated for Cancer Prevention and Treatment, Tongde Hospital of Zhejiang Province, Hangzhou, Zhejiang, China; d Zhejiang Provincial Key Laboratory of Traditional Chinese Medicine for Pharmacodynamic Material Basis Research of Chinese Medicine, Tongde Hospital of Zhejiang Province Affiliated to Zhejiang Chinese Medical University, Hangzhou, Zhejiang, China; e Department of Infection Prevention and Control, Tongde Hospital of Zhejiang Province, Hangzhou, Zhejiang, China.

**Keywords:** cefuroxime, FAERS, hepatobiliary disorders, immune system disorders, JADER, skin and subcutaneous tissue disorders

## Abstract

Cefuroxime, as a second-generation cephalosporin, is widely used in clinical practice. In recent years, research using the Food and Drug Administration Adverse Event Reporting System (FAERS) database has led to new discoveries about the adverse event profile of cefuroxime. However, there is currently a lack of systematic research on the safety of cefuroxime based on the Japan Adverse Drug Event Report (JADER) database. This study analyzed the clinical characteristics and adverse event signals associated with cefuroxime using the JADER database and further compared the findings with those from the FAERS database. A descriptive analysis was conducted on the clinical characteristics of adverse event reports associated with cefuroxime, including report year, report season, onset time, reporter type, sex, age, weight, dose, frequency, and indication. Four disproportionality algorithms were employed to identify adverse event signals. Subsequently, the clinical characteristics and disproportionality signals of cefuroxime in the JADER database were contrasted with those in the FAERS database. A total of 60 adverse event reports and 132 adverse events related to cefuroxime were identified from the JADER database. “Oculomucocutaneous syndrome” emerged as a newly identified disproportionality signal in the JADER database, which was neither reported in the FAERS database nor previously documented in cefuroxime’s labeling. “Anaphylactic shock,” “drug-induced liver injury,” “jaundice,” and “Stevens-Johnson syndrome” were identified as disproportionality signals in both the JADER and FAERS databases. “Anaphylactic shock” had a stronger signal in the FAERS database, and “drug-induced liver injury” and “jaundice” exhibited stronger signals in the JADER database. This study provides a descriptive analysis and signal detection using the JADER database, contributing to previous research based solely on the FAERS database and offering an additional perspective on the clinical safety profile of cefuroxime. This finding suggests a potential need for enhanced monitoring for “skin and subcutaneous tissue disorders,” “immune system disorders,” and “hepatobiliary disorders.” It should be noted that the results from the JADER and FAERS databases may be influenced by reporting biases. Therefore, further studies are warranted to corroborate the findings.

**Key Points**

- “Oculomucocutaneous syndrome” emerged as a novel adverse event signal.
- “Anaphylactic shock” showed a stronger signal in the FAERS database.
- “Drug-induced liver injury” and “jaundice” exhibited stronger signals in the JADER database.

## 1. Introduction

Cefuroxime is a second-generation cephalosporin antibiotic with broad-spectrum antimicrobial activity, enabling it to effectively combat a variety of bacterial infections.^[1]^ It exerts its effect by inhibiting the catalytic activity of bacterial transpeptidases, causing gradual dissolution and ultimately death of the bacteria.^[2]^ Over the past decades, cefuroxime has been widely utilized in the treatment of a variety of infectious conditions, such as respiratory tract infections, genitourinary infections, and skin and soft tissue infections.^[3]^

The spontaneous reporting database serves as a vital tool for pharmacovigilance research.^[4]^ The Food and Drug Administration Adverse Event Reporting System (FAERS) is the largest global database for monitoring drug-related adverse events.^[5]^ It has been instrumental in evaluating the post-marketing safety of various antibiotics, such as cefotaxime, tigecycline, ceftazidime/avibactam, and piperacillin/tazobactam.^[6–9]^ In recent years, research using the FAERS database has led to new discoveries about the adverse events of cefuroxime. Teng et al found a statistically significant increased risk of delirium with cefuroxime.^[10]^ Yan et al demonstrated that cefuroxime exhibited significant associations with Stevens-Johnson syndrome and toxic epidermal necrolysis.^[11]^ Furthermore, our previous research^[12]^ and Li et al^[13]^ found that cefuroxime was reported to pose a risk of drug-induced eye disorders. Despite cefuroxime having been on the market for decades, it is still necessary to continuously conduct systematic research on its post-marketing adverse events profile.

Although the FAERS database has been used for monitoring cefuroxime-related adverse events, it has certain limitations. For instance, FAERS’ adverse event reports are mainly from the United States. In addition, adverse event reports submitted by nonprofessionals, such as consumers, may be subject to certain biases. It is necessary to further explore the post-marketing adverse events of cefuroxime through studies involving multiple databases. The Japan Adverse Drug Event Report (JADER) database is an adverse event reporting database developed by the Pharmaceutical and Medical Device General Organization.^[14]^ Compared to the FAERS database, the JADER database primarily contains reports from Asia, and the reports are mainly submitted by healthcare professionals.^[15]^ It has been applied in safety evaluation studies of drugs such as memantine and ganciclovir.^[16,17]^ However, there is currently a lack of systematic research on the safety of cefuroxime based on the JADER database.

This study investigated the clinical characteristics and adverse event signals of cefuroxime based on the JADER database and further compared the findings with those from the FAERS database. The results of this study contribute to previous research based solely on the FAERS database and offer additional perspectives on the clinical safety profile of cefuroxime. As spontaneous reporting systems, both databases are subject to inherent limitations such as reporting biases and incomplete data. Additional studies are warranted to validate the findings.

## 2. Materials and methods

### 2.1. Data source and collection

The data covered the period from April 2004 to July 2024 in the JADER database. All data were sourced from the Pharmaceuticals and Medical Devices Agency website (www.pmda.go.jp). Ethical approval was not required because this study used the JADER database, which is a free open-access database. Duplicate reports were removed from the files. Reports of cefuroxime were identified by searching for the generic name as the suspected drug. A descriptive analysis was conducted on the clinical characteristics of adverse event reports associated with cefuroxime, including report year, report season, onset time, reporter type, sex, age, weight, dose, frequency, and indication. The standardized Medical Dictionary for Regulatory Activities version 27.0 (approved by the International Council for Harmonisation of Technical Requirements for Pharmaceuticals for Human Use) was utilized to classify the preferred term (PT) into the corresponding primary system organ class (SOC) levels. For more information on the data source and collection process, please refer to the reference.^[18]^

### 2.2. Statistical analysis

Four disproportionality algorithms were employed to identify adverse event signals associated with cefuroxime at both the SOC and PT levels. These 4 algorithms included reporting odds ratio,^[19,20]^ proportional reporting ratio,^[21,22]^ Bayesian confidence propagation neural network,^[20–22]^ and multi-item gamma Poisson shrinker.^[20–22]^ The adverse events for cefuroxime were compared with those for all other drugs. Reports with missing clinical characteristic data were excluded from the respective clinical characteristics but were retained in the signal detection. The clinical characteristics and signal strengths of cefuroxime in the JADER database were contrasted with those in the FAERS database. For more detailed results from the FAERS database and further specifics on the statistical analysis, please refer to our previous articles.^[12]^ Data processing and statistical analyses were conducted using the Python 3 programming language within Jupyter Notebook version 6.4.12. It should be noted that the results from the JADER and FAERS databases may be influenced by reporting biases. Furthermore, as spontaneous reporting systems, both databases are subject to inherent limitations such as incomplete data, which may predispose the results to bias. Therefore, further studies are warranted to corroborate the findings.

## 3. Results

### 3.1. Clinical characteristics

**Figure 1.**
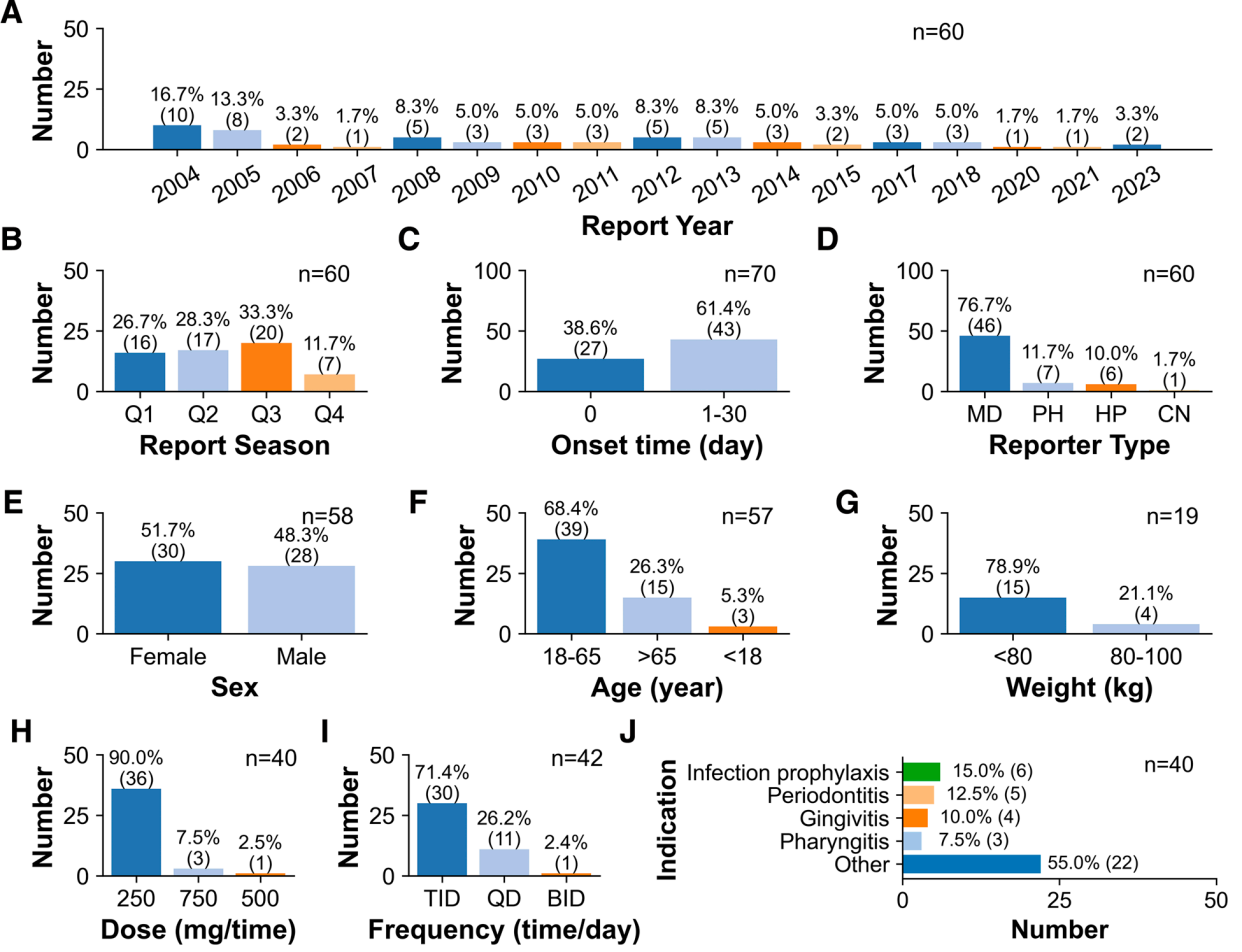
Clinical characteristics of cefuroxime-associated adverse event reports from the JADER database. (A) Report year. (B) Report season. (C) Onset time. (D) Reporter type. (E) Sex. (F) Age. (G) Weight. (H) Dose. (I) Frequency. (J) Indication. BID = twice a day, CN = consumer, HP = health professional, JADER = Japan Adverse Drug Event Report, MD = physician, PH = pharmacist, QD = once a day, TID = 3 times a day.

A total of 60 adverse event reports and 132 adverse events related to cefuroxime were identified from the JADER database. Figure 1 illustrates the clinical characteristics of cefuroxime-related adverse event reports from the JADER database. The third quarter reported the highest number of reports, consistent with findings in the FAERS database, which may be attributed to factors such as the increased incidence of infectious diseases or heightened susceptibility to allergic reactions during this period. All adverse events occurred within 30 days of administration, with 38.6% on the day of medication and 61.4% within 1 to 30 days. This alignment with FAERS results suggests the importance of monitoring within the initial 30 days of treatment.

In this study, 76.7% of reports were from physicians, followed by pharmacists (11.7%), health professionals (10.0%), and consumers (1.7%). The predominance of professional reporters in the JADER database, in contrast to consumer reporters in the FAERS database, underscores the reliability of the study data. Reports related to females slightly outnumbered those related to males, with 51.7% females and 48.3% males. However, in the FAERS database, the proportion of adverse event reports in females significantly exceeded that in males (female vs male, 61.8% vs 38.2%). Further research is warranted to explore whether this discrepancy is due to differences in demographics.

Majority of individuals were aged between 18 and 65 years (68.4%) and weighed <80 kg (78.9%), consistent with FAERS results. In the JADER database, all reports involved oral administration of tablets. The most common dose and frequency were 250 mg (90.0%) and 3 times a day (71.4%). In the FAERS database, the most common oral dose and frequency of tablets were 500 mg and twice daily. These distinctions may be influenced by variations in the indications for cefuroxime between the 2 databases. In the JADER database, the most frequently reported indications included “infection prevention,” “periodontitis,” “gingivitis,” and “pharyngitis.” In comparison, the most common indications for cefuroxime administration in FAERS were “antibiotic prophylaxis,” “urinary tract infection,” “prophylaxis,” and “sinusitis.”

### 3.2. Signals at the SOC level

In the JADER database, adverse event reports linked to cefuroxime encompassed 13 SOCs. Table 1 displays the number and signal strength of cefuroxime at the SOC level from the JADER database. “Skin and subcutaneous tissue disorders” and “immune system disorders” were SOCs that met the criteria of 4 disproportionality algorithms. “Hepatobiliary disorders,” ranked second in terms of reported numbers, met the criteria of 3 algorithms.

**Table 1:** Number and signal strength of cefuroxime at the SOC level from the JADER database.

SOC	Number	ROR (95% CI)	PRR (χ^2^)	IC (IC025)	EBGM (EBGM05)
Skin and subcutaneous tissue disorders (SOC: 10040785)*,†,‡,§	32	4.73 (3.18–7.05)	3.83 (71.35)	1.94 (1.25)	3.83 (2.57)
Hepatobiliary disorders (SOC: 10019805)*,†,‡	18	3.31 (2.01–5.44)	2.99 (25.01)	1.58 (0.72)	2.99 (1.82)
Immune system disorders (SOC: 10021428)*,†,‡,§	16	5.07 (3.01–8.55)	4.58 (45.91)	2.19 (1.17)	4.57 (2.71)
Nervous system disorders (SOC: 10029205)	12	1.08 (0.60–1.95)	1.07 (0.06)	0.10 (−0.76)	1.07 (0.59)
Gastrointestinal disorders (SOC: 10017947)	10	0.90 (0.47–1.72)	0.91 (0.10)	−0.14 (−1.04)	0.91 (0.48)
General disorders and administration site conditions (SOC: 10018065)	10	1.30 (0.68–2.47)	1.28 (0.64)	0.35 (−0.60)	1.28 (0.67)
Investigations (SOC: 10022891)	10	0.72 (0.38–1.37)	0.74 (1.00)	−0.43 (−1.31)	0.74 (0.39)
Respiratory, thoracic, and mediastinal disorders (SOC: 10038738)	10	1.10 (0.58–2.10)	1.09 (0.08)	0.13 (−0.80)	1.09 (0.57)
Vascular disorders (SOC: 10047065)	5	1.62 (0.66–3.95)	1.59 (1.13)	0.67 (−0.68)	1.59 (0.65)
Infections and infestations (SOC: 10021881)	4	0.31 (0.11–0.83)	0.33 (6.03)	−1.60 (−2.72)	0.33 (0.12)
Ear and labyrinth disorders (SOC: 10013993)*	2	8.00 (1.98–32.34)	7.89 (12.06)	2.98 (−0.43)	7.89 (1.95)
Musculoskeletal and connective tissue disorders (SOC: 10028395)	2	0.63 (0.16–2.54)	0.63 (0.43)	−0.66 (−2.16)	0.63 (0.16)
Renal and urinary disorders (SOC: 10038359)	1	0.19 (0.03–1.34)	0.19 (3.51)	−2.37 (−3.69)	0.19 (0.03)

BCPNN = Bayesian confidence propagation neural network, CI = confidence interval, EBGM = empirical Bayesian geometric mean, EBGM05 = lower limit of 95% confidence interval of EBGM, IC = information component, IC025 = lower limit of 95% confidence interval of IC, JADER = Japan Adverse Drug Event Report, PRR = proportional reporting ratio, ROR = reporting odds ratio, SOC = system organ class, χ^2^ = chi-squared.

* SOCs met the criteria of ROR algorithm.

† SOCs met the criteria of PRR algorithm.

‡ SOCs met the criteria of BCPNN algorithm.

§ SOCs met the criteria of MGPS algorithm.

**Figure 2.**
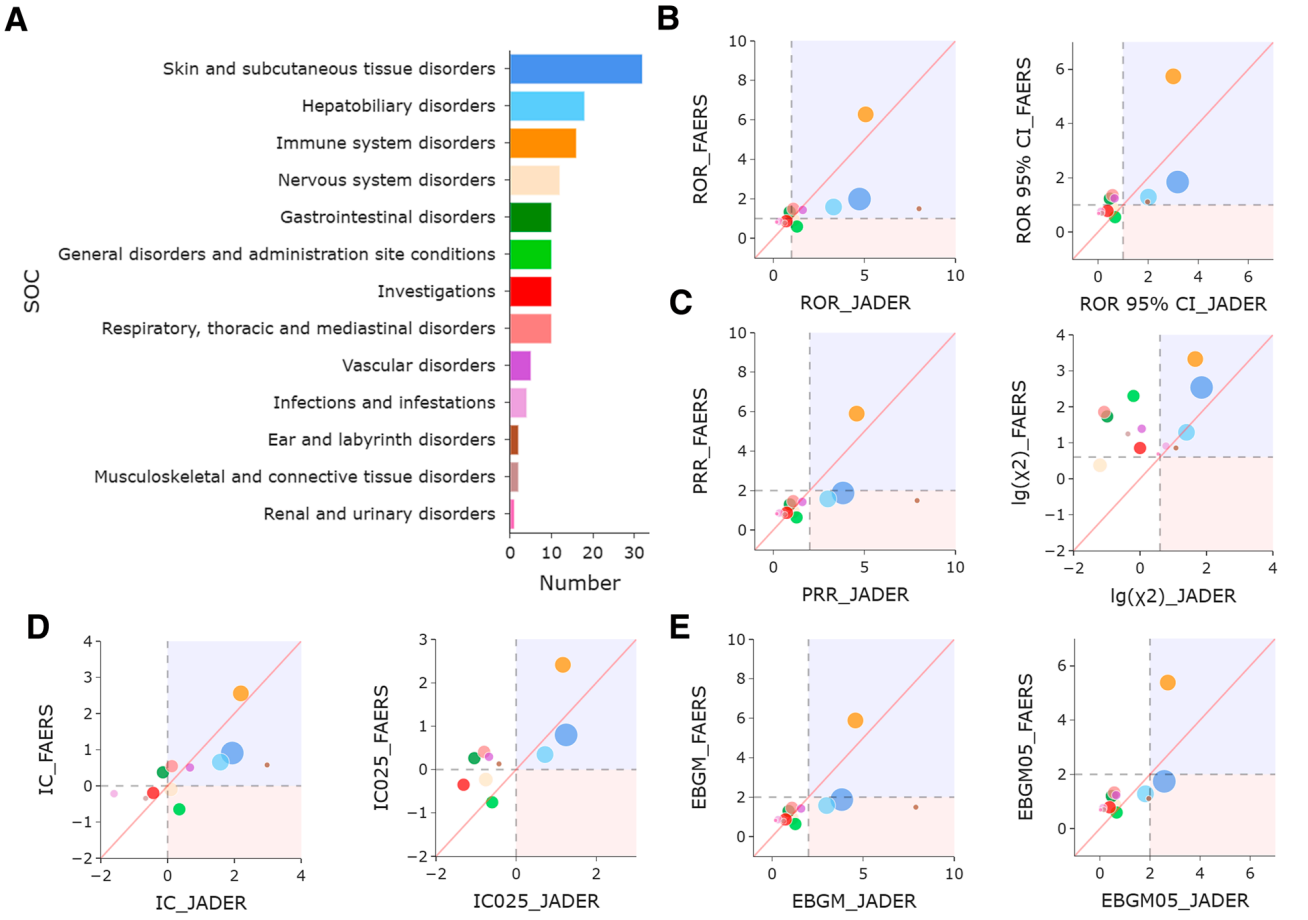
Signals associated with cefuroxime at the SOC level. (A) Number and color representation of 13 SOCs in the JADER database. (B) Signal strength in both the JADER and FAERS databases based on ROR algorithms. (C) Signal strength in both the JADER and FAERS databases based on PRR algorithms. (D) Signal strength in both the JADER and FAERS databases based on BCPNN algorithms. (E) Signal strength in both the JADER and FAERS databases based on MGPS algorithms. BCPNN = Bayesian confidence propagation neural network, CI = confidence interval, EBGM = empirical Bayesian geometric mean, EBGM05 = lower limit of 95% confidence interval of EBGM, FAERS = Food and Drug Administration Adverse Event Reporting System, IC = information component, IC025 = lower limit of 95% confidence interval of IC, JADER = Japan Adverse Drug Event Report, PRR = proportional reporting ratio, ROR = reporting odds ratio, SOC = system organ class, χ^2^ = chi-squared.

Compared to the FAERS database, which involved 27 SOCs associated with cefuroxime, adverse event reports in the JADER database were more concentrated. Figure 2A illustrates the number and color representation of 13 SOCs associated with cefuroxime in the JADER database. Figure 2B–E present the signal strength of cefuroxime at the SOC level in both the JADER and FAERS databases based on 4 disproportionality algorithms. The “immune system disorders” was the only SOC that met the criteria of 4 disproportionality algorithms in both the JADER and FAERS databases. “Skin and subcutaneous tissue disorders” met the criteria of 4 algorithms in the JADER database and only 2 algorithms in the FAERS database. “Hepatobiliary disorders” met at least 1 algorithm in both the JADER and FAERS databases. The SOCs of “eye disorders” and “pregnancy, puerperium, and perinatal conditions,” which simultaneously met the criteria of 4 disproportionality algorithms in the FAERS database, were not disproportional in the JADER database.

### 3.3. Signals at the PT level

A total of 7 disproportionality PTs associated with cefuroxime were detected from the JADER database, including “anaphylactic shock,” “drug-induced liver injury,” “Stevens-Johnson syndrome,” “drug eruption,” “jaundice,” “oculomucocutaneous syndrome,” and “dizziness.” Table 2 displays the number and signal strength of cefuroxime at the PT level from the JADER database. Notably, “oculomucocutaneous syndrome” emerged as a newly identified disproportionality signal in the JADER database, which was neither reported in the FAERS database nor previously documented in cefuroxime’s labeling.

**Table 2:** Number and signal strength of cefuroxime at the PT level from the JADER database.

PT	Number	ROR (95% CI)	PRR (χ^2^)	IC (IC025)	EBGM (EBGM05)
Skin and subcutaneous tissue disorders (SOC: 10040785)					
Stevens-Johnson syndrome (PT: 10042033)	7	10.09 (4.71–21.61)	9.61 (54.27)	3.26 (1.15)	9.61 (4.49)
Drug eruption (PT: 10013687)	6	6.44 (2.84–14.61)	6.19 (26.30)	2.63 (0.71)	6.19 (2.73)
Oculomucocutaneous syndrome (PT: 10030081)	3	28.15 (8.95–88.51)	27.54 (76.67)	4.78 (0.38)	27.50 (8.75)
Hepatobiliary disorders (SOC: 10019805)					
Drug-induced liver injury (PT: 10072268)	7	11.80 (5.51–25.26)	11.22 (65.46)	3.49 (1.25)	11.22 (5.24)
Jaundice (PT: 10023126)	4	19.05 (7.04–51.56)	18.50 (66.27)	4.21 (0.72)	18.49 (6.83)
Immune system disorders (SOC: 10021428)					
Anaphylactic shock (PT: 10002199)	11	9.33 (5.03–17.29)	8.63 (74.91)	3.11 (1.52)	8.63 (4.65)
Nervous system disorders (SOC: 10029205)					
Dizziness (PT: 10013573)	3	7.27 (2.31–22.85)	7.13 (15.85)	2.83 (0.03)	7.13 (2.27)

CI = confidence interval, EBGM = empirical Bayesian geometric mean, EBGM05 = lower limit of 95% confidence interval of EBGM, IC = information component, IC025 = lower limit of 95% confidence interval of IC, JADER = Japan Adverse Drug Event Report, PRR = proportional reporting ratio, PT = preferred term, ROR = reporting odds ratio, χ^2^ = chi-squared.

**Figure 3.**
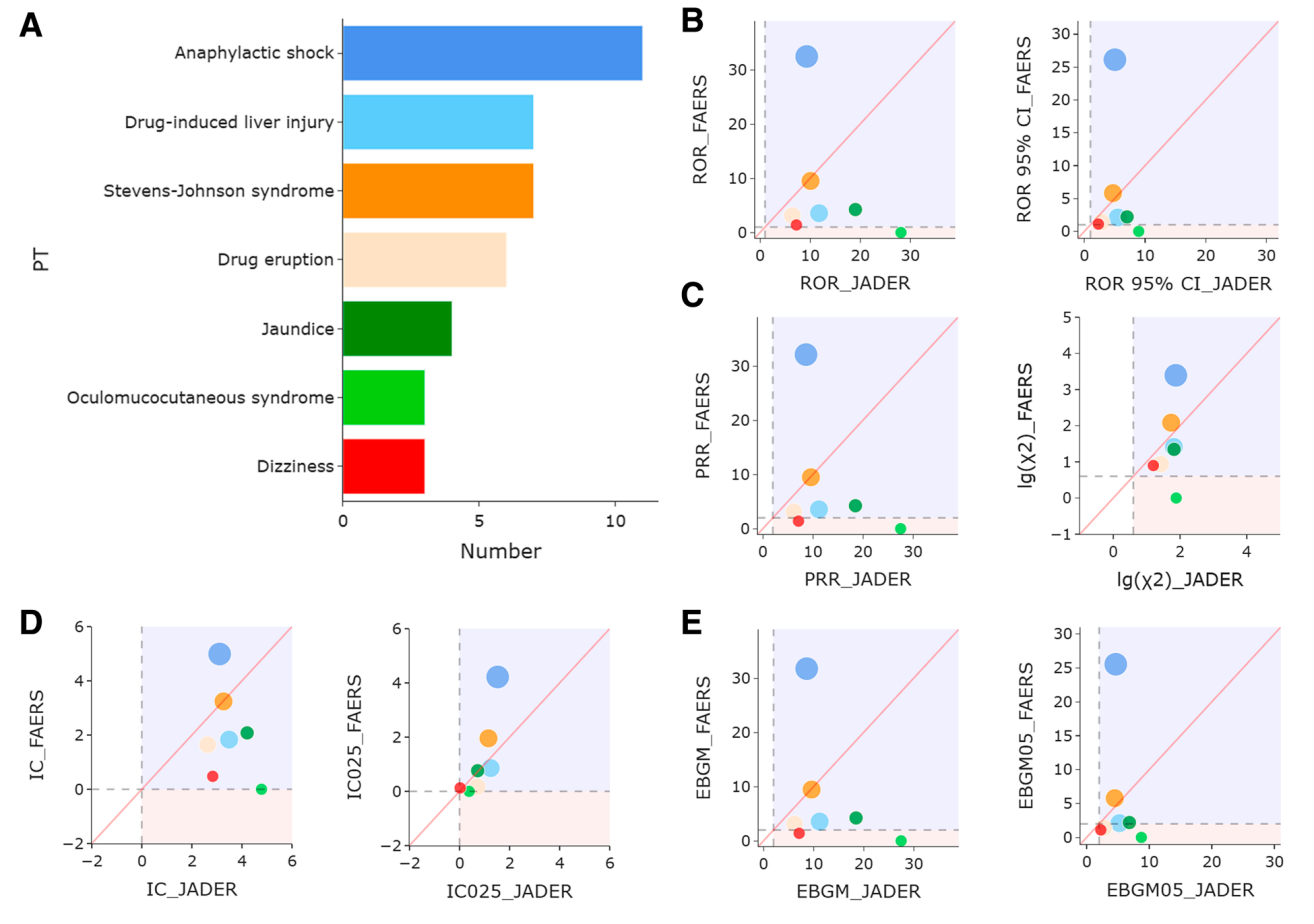
Signals associated with cefuroxime at the PT level. (A) Number and color representation of 7 PTs in the JADER database. (B) Signal strength in both the JADER and FAERS databases based on ROR algorithms. (C) Signal strength in both the JADER and FAERS databases based on PRR algorithms. (D) Signal strength in both the JADER and FAERS databases based on BCPNN algorithms. (E) Signal strength in both the JADER and FAERS databases based on MGPS algorithms. BCPNN = Bayesian confidence propagation neural network, CI = confidence interval, EBGM = empirical Bayesian geometric mean, EBGM05 = lower limit of 95% confidence interval of EBGM, FAERS = Food and Drug Administration Adverse Event Reporting System, IC = information component, IC025 = lower limit of 95% confidence interval of IC, JADER = Japan Adverse Drug Event Report, PRR = proportional reporting ratio, PT = preferred term, ROR = reporting odds ratio, χ² = chi-squared.

Figure 3A displays the number and color representation of 7 cefuroxime-associated PTs in the JADER database. The signal strength of cefuroxime at the PT level in both the JADER and FAERS databases is illustrated in Figure 3B–E. The PTs “anaphylactic shock,” “drug-induced liver injury,” “jaundice,” and “Stevens-Johnson syndrome” met the criteria of 4 disproportionality algorithms in both the JADER and FAERS databases. Among these, “anaphylactic shock” had a stronger signal in the FAERS database. Conversely, “drug-induced liver injury” and “jaundice” exhibited stronger signals in the JADER database. “Stevens-Johnson syndrome” had similar signal strengths in 2 databases. This finding suggests potential need for enhanced monitoring for “skin and subcutaneous tissue disorders” (e.g., “oculomucocutaneous syndrome” and “Stevens-Johnson syndrome”), “immune system disorders” (e.g., “anaphylactic shock”), and “hepatobiliary disorders” (e.g., “drug-induced liver injury” and “jaundice”).

## 4. Discussion

### 4.1. Skin and subcutaneous tissue disorders

Through the application of 4 disproportionality algorithms, a disproportionality SOC of “skin and subcutaneous tissue disorders” was identified from the JADER database. Under the “skin and subcutaneous tissue disorders” category, 3 disproportionality signals at the PT level were detected, including “Stevens-Johnson syndrome,” “drug eruption,” and “oculomucocutaneous syndrome.” Previous study also revealed a disproportionality adverse event signal for “Stevens-Johnson syndrome” in FAERS database, emphasizing the importance of clinicians remaining vigilant. “Stevens-Johnson syndrome” is a rare yet severe drug-induced skin adverse reaction characterized by widespread skin and mucosal lesions, with a global mortality rate ranging from 34% to 50%.^[23,24]^ Statistics indicate that approximately 80% of cases associated with this syndrome involve antibiotics, anticonvulsants, allopurinol, or analgesics.^[25,26]^ The onset of the reaction can be rapid, occurring within 4 days of drug administration, with a higher incidence seen 4 to 8 weeks post-use.^[27]^ Complications such as sepsis and multiple organ failure may accompany the syndrome, potentially leading to patient mortality.^[28]^ During treatment, regular evaluation of the patient’s skin, mucous membranes, renal function, and visual system is advised to promptly diagnose the syndrome and its potential complications.^[29]^ If diagnosed, immediate discontinuation of the offending drug and administration of corticosteroids and intravenous immunoglobulin are recommended for treatment.^[30]^

Notably, “oculomucocutaneous syndrome” emerged as a new disproportionality adverse event signal, which was neither reported in the FAERS database nor previously documented in cefuroxime’s labeling. This syndrome affects multiple organs, with patients potentially experiencing skin rashes, hearing loss, dry eyes, as well as pleural and peritoneal effusions.^[31]^ Studies suggest that the underlying pathogenesis might involve an immune-mediated skin disease affecting the eyes and other mucosal surfaces.^[32]^ While oculomucocutaneous syndrome shares similar clinical manifestations with Stevens-Johnson syndrome, including erythema, rash, and skin and mucous membrane peeling, they can be distinguished based on early ocular and non-ocular symptoms.^[32]^ The distinguishing feature of oculomucocutaneous syndrome lies in its ability to not only affect the patient’s skin and mucous membranes but also lead to reduced tear secretion, resulting in severe dry eye.^[31]^ Given the severity of oculomucocutaneous syndrome, this study recommends further in-depth investigation for exploring the association between oculomucocutaneous syndrome and cefuroxime.

### 4.2. Immune system disorders

The “immune system disorders” was the only SOC that met the criteria of 4 disproportionality algorithms in both the JADER and FAERS databases. “Anaphylactic shock” was detected as the sole disproportionality signal under the “immune system disorders” category in JADER database. Anaphylactic shock is a severe systemic hypersensitivity reaction that occurs rapidly and can potentially lead to sudden death. The signal for anaphylactic shock was stronger in the FAERS database compared to JADER. The influencing factors for this difference are complex, such as potential ethnic differences, reporting biases between the 2 databases, and differences in formulations. Patients with a history of penicillin or cephalosporin allergy have been more prone to developing serious immune system diseases.^[33,34]^ Obesity can also increase the risk of immune-related diseases.^[35]^ Furthermore, the FAERS database included a wider range of cefuroxime formulations, such as tablets and injections, with administration routes including oral and intravenous, whereas the JADER database only had oral cefuroxime tablets. Our previous research has shown that intravenous administration is more likely to lead to anaphylactic shock compared to oral.^[12]^ To eliminate the impact of administration routes, we further conducted signal detection specifically for oral cefuroxime in the FAERS database, and the results still showed a stronger signal for anaphylactic shock compared to the JADER database (FAERS, oral cefuroxime, reporting odds ratio [95% CI]: 21.47 [12.91–35.71]; proportional reporting ratio [χ^2^]: 21.31 [290.01]; IC [IC025]: 4.41 [2.50]; EBGM [EBGM05]: 21.28 [12.80]). To some extent, these findings rule out the influence of formulation, but they cannot eliminate the impact of reporting biases between the 2 databases. Therefore, further studies are needed to verify whether there may be potential ethnic differences in the occurrence of anaphylactic shock.

### 4.3. Hepatobiliary disorders

“Hepatobiliary disorders” ranked second in the number of reports and were identified by 3 signal detection algorithms. This study found that “drug-induced liver injury” and “jaundice” were disproportionality hepatobiliary disorders in both the JADER and FAERS databases. Moreover, “drug-induced liver injury” and “jaundice” exhibited stronger signals in the JADER database, which warrants attention. Drug-induced liver injury is a serious clinical issue and represents one of the most challenging types of liver disease. Research has shown that antibiotics are a common cause of drug-induced liver injury.^[36]^ High concentrations of cefuroxime may lead to hepatocyte damage due to drug accumulation.^[37]^ In addition, drug-induced liver injury may result from immune-mediated responses or reactions to hepatotoxic metabolites.^[38]^ Recent studies have highlighted the close association between antibiotic-induced liver injury and the intestinal microbiota, which may contribute to liver injury through mechanisms such as increased intestinal permeability, disruption of metabolic homeostasis, and promotion of inflammation and oxidative stress.^[39,40]^ The findings suggest that clinicians should remain vigilant for potential liver injury associated with cefuroxime.

### 4.4. Strengths and limitations

This study investigated the clinical characteristics and adverse event signals associated with cefuroxime using the JADER database and further compared the findings with those from the FAERS database, revealing several key insights. First, “skin and subcutaneous tissue disorders” was detected as the disproportionality SOC in the JADER database, but was not disproportionality in the FAERS database. Under this SOC, “oculomucocutaneous syndrome” emerged as a newly identified disproportionality signal, which was neither reported in the FAERS database nor previously documented in cefuroxime’s labeling. Second, “immune system disorders” was the only SOC that met the criteria of 4 algorithms in both the JADER and FAERS database. Under this SOC, “anaphylactic shock” showed a stronger signal in the FAERS database. Third, hepatobiliary disorders including “drug-induced liver injury” and “jaundice” exhibited stronger signals in the JADER database. To the best of our knowledge, this represents the first comprehensive pharmacovigilance study to systematically evaluate cefuroxime-associated adverse events using the JADER database and to compare the results with those from the FAERS database.

Several limitations should be noted. First, the sample size of the JADER database is relatively smaller than that of the FAERS database. Second, the results from the JADER and FAERS databases may be influenced by reporting biases, underreporting, and differences in healthcare systems between Japan and the United States, as these factors can influence signals and comparability. Third, the concurrent use of traditional Chinese medicines, which are involved in the regulation of liver and immune functions,^[41–43]^ was not considered in this study. Fourth, the novel signal for oculomucocutaneous syndrome detected exclusively in JADER may partly reflect differential awareness or coding practices for this adverse event in Japan, rather than a true population-level difference in risk. Therefore, the association between oculomucocutaneous syndrome and cefuroxime requires further in-depth investigation. Despite these limitations, by analyzing data from the JADER database, this study contributes to previous research based solely on the FAERS database and offers additional perspective on the clinical safety profile of cefuroxime.

## 5. Conclusions

This study provides a descriptive analysis and signal detection using the JADER database. “Oculomucocutaneous syndrome” emerged as a newly identified disproportionality signal in the JADER database, which was neither reported in the FAERS database nor previously documented in cefuroxime’s labeling. “Anaphylactic shock” had a stronger signal in the FAERS database, and “drug-induced liver injury” and “jaundice” exhibited stronger signals in the JADER database. This finding suggests potential need for enhanced monitoring for “skin and subcutaneous tissue disorders,” “immune system disorders,” and “hepatobiliary disorders.” Additional studies are warranted to validate these findings.

## Acknowledgments

We would like to thank the participants and researchers of the JADER database. All authors confirm that this manuscript is our original work and has not been published nor has it been submitted simultaneously elsewhere. All authors have checked the manuscript and have agreed to the submission.

## Author contributions

**Formal analysis:** Fangqiong Li.

**Writing – original draft:** Fangqiong Li, Jinyu Luo, Xinhao Zhang.

**Conceptualization:** Cheng Jiang, Guoqin Xia.

**Writing – review & editing:** Cheng Jiang, Guoqin Xia.
